# The Role of Noncoding RNA in the Pathophysiology and Treatment of Premature Ovarian Insufficiency

**DOI:** 10.3390/ijms22179336

**Published:** 2021-08-28

**Authors:** Katarzyna Pankiewicz, Piotr Laudański, Tadeusz Issat

**Affiliations:** 1 Department of Obstetrics and Gynecology, Institute of Mother and Child in Warsaw, Kasprzaka 17a, 01-211 Warsaw, Poland; tadeusz.issat@imid.med.pl; 2 1st Department of Obstetrics and Gynecology, Medical University of Warsaw, Starynkiewicza 1/3, 02-015 Warsaw, Poland; piotr.laudanski@wum.edu.pl; 3 OVIklinika Infertility Center, Połczyńska 31, 01-377 Warsaw, Poland

**Keywords:** premature ovarian insufficiency, noncoding RNA, microRNA, mesenchymal stem cells, infertility

## Abstract

Premature ovarian insufficiency (POI) is defined as a loss of ovarian function before the age of 40 years, with a prevalence rate estimated at approximately 1%. It causes infertility and is related to serious long-term health consequences, including reduced life expectancy, increased cardiovascular risk, decreased bone mineral density and neurological disorders. There is currently no effective therapy for POI that is widely available in clinical practice; therefore, the treatment of patients with POI is based on hormone replacement therapy. One of the recent advances in the understanding of the pathophysiology of POI has been the role of microRNAs (miRNAs) and other noncoding RNAs (ncRNAs) in the disease. Moreover, intensive research on human folliculogenesis and reproductive biology has led to the development of novel promising therapeutic strategies with the use of exosomal miRNAs derived from mesenchymal stem cells to restore ovarian function in POI patients. This narrative review focuses on the new studies concerning the role of ncRNAs in the pathogenesis of POI, together with their potential as biomarkers of the disease and targets for therapy.

## 1. Introduction

Premature ovarian insufficiency (POI) is defined as a loss of ovarian function before the age of 40 years. It was first described by Fuller Albright in 1942, and since that time, different terms have been used for this condition, such as premature ovarian failure (POF), premature menopause, early menopause, primary ovarian failure and primary ovarian insufficiency [1,2]. According to the Guidelines of the European Society of Human Reproduction and Embryology (ESHRE), POI is diagnosed by meeting the following criteria: amenorrhea or oligomenorrhea for at least four months and elevated follicle stimulating hormone (FSH) level > 25 IU/L on two occasions at least four weeks apart [3].

The prevalence of POI is estimated at approximately 1%; however, in some ethnic groups it may be higher, e.g., 2.8% in Chinese women [4,5]. Clinical symptoms of POI are related to hypoestrogenism and may include amenorrhea, oligomenorrhea, hot flushes, night sweats, vulvovaginal atrophy, sleep disturbances and irritability. Loss of ovarian function causes infertility, with a very low chance of spontaneous pregnancy (about 5%) [6]. Moreover, POI has serious long-term sequelae for women’s health, including reduced life expectancy, increased cardiovascular risk, decreased bone mineral density and impacts on neurological health (possible detrimental effects on cognition) [3]; therefore, patients with POI should receive special multidisciplinary care to avoid these consequences.

Research concerning various mechanisms of POI pathophysiology may help find specific targets for both the effective treatment of infertility and prevention of the long-term complications in this group of patients. One of the recent advances in the pathophysiology of POI has been the elucidation of the role of microRNAs (miRNA) and other noncoding RNAs (ncRNA), giving an opportunity to develop new treatment strategies. In this narrative review, we focus on the new studies concerning the role of miRNAs in the pathogenesis of POI, together with their potential as biomarkers of the disease and targets for therapy.

## 2. Noncoding RNAs

It is estimated that protein-coding sequences in humans account for only 1.5% of the entire genome. The term ncRNA refers to RNA molecules that cannot translate proteins but have important regulatory functions in many biological processes, such as cell proliferation and adhesion, apoptosis, angiogenesis and migration [7,8]. NcRNAs can be divided by their length into small ncRNAs (sncRNAs), consisting of up to 200 nucleotides, and long ncRNAs (lncRNA), containing more than 200 nucleotides.

SncRNAs include miRNAs, small nucleolar RNAs (snoRNAs), small nuclear RNAs (snRNAs), small interfering RNAs (siRNAs), transfer RNAs (tRNAs) and piwi-interacting RNAs (piRNAs) [9]. MiRNAs are well-known and are the most studied class of sncRNAs. Each miRNA consists of 21–22 nucleotides and is formed from a miRNA precursor with a hairpin ring structure through the activities of the Drosha and Dicer ribonuclease III endonucleases. They suppress protein expression by silencing messenger RNA (mRNA) translation and causing target mRNA degradation. The seed region of an miRNA is complementary to the 3′ untranslated region (3′ UTR) of the target gene to prevent transcription [10]. MiRNAs are conserved evolutionarily and show tissue specificity [11]. SiRNAs suppress gene expression through a highly regulated enzyme-mediated process called RNA interference (RNAi) and are very promising as a potent targeted therapeutic modality with applications ranging from viral diseases to cancer [12,13]. PiRNAs are larger than miRNAs (consisting of 26–31 nucleotides), their processing does not require Dicer and they bind to a specific class of Ago proteins known as P-Element induced wimpy testis (PIWI) proteins. PiRNAs are found almost exclusively in germ cells, so they may have an important role in female reproductive diseases [14,15]; however, piRNA and PIWIprotein expression has recently been reported in some human cancers, with some piRNA–piwi complexes participating in the neoplasm pathogenesis and associated with cancer prognosis [16].

LncRNAs include long-intron ncRNAs, long intergenic ncRNAs (lincRNAs) and circular RNAs (circRNAs). Most of these are located in the nucleus and act as molecular scaffolds in alternative splicing and modification of the chromatin structure. Some lncRNAs may also function in the cytoplasm in regulating translation, mRNA degradation and as miRNA sponges. These miRNA sponges are RNAs that contain complementary binding sites to an miRNA of interest, and are produced from transgenes within cells. The use of such miRNA sponges is a transgenic approach that has proven to be a useful in probing miRNA functions in a variety of experimental systems [17,18,19,20]. The most studied class of lncRNA comprises circRNAs, which have no free 3′ or 5′ end after reverse splicing and may act as miRNA sponges, protein sponges, nuclear transcriptional regulators and protein scaffolds [21,22]. Recent studies have revealed that circRNAs are dysregulated in multiple human disorders, such as cancer, cardiovascular and neurological diseases [23,24,25,26].

## 3. Pathogenesis of POI

There is a wide range of possible causes of POI; however, approximately 90% of POI cases remain idiopathic. Among the known etiologies of POI are genetics, autoimmune diseases, environmental factors and iatrogenic reasons [3,27,28].

In recent years, the genetic causes of POI have been intensively studied. Thanks to genome-wide association studies (GWAS), whole-exome sequencing (WES) and next-generation sequencing (NGS techniques), together with the evaluation of gene copy variants and family-based studies, a variety of genes crucial for ovarian function and senescence have been identified [29]. It is estimated that around 7–25% of POI cases are genetically determined [27,30]. The best-known genetic abnormalities associated with POI are related to the X chromosome and include Turner syndrome (45,X) and fragile X syndrome (FMR1 mutation). The partial insufficiency of many genes located on the X chromosome in Turner syndrome leads to oocyte apoptosis and oocyte depletion within the first 10 years of life; however, the degree of mosaicism contributes to the phenotypic variation in this syndrome [31,32]. Women with the FMR1 premutation have increased risk of POI; however, this depends on the number of repeats of CGG trinucleotide in the 5′ end of the FMR1 gene. Expansion to more than 200 repeats leads to fragile X syndrome associated with mental retardation. The risk of POI is highest with 80–100 repeats and decreases with more than 100. Importantly, even within the healthy range of repeat size length, longer repeats are more likely to cause an advanced stage of ovarian ageing than shorter repeats [33,34].

So far, the discovered genes associated with POI can be sorted into functional categories. Mutations in various reproductive ligands and their receptors include FSH receptor mutation (FSHR), bone morphogenic protein 15 (BMP15) and its paralog growth differentiation factor 9 (GDF9) mutations, gremlin 1 (GREM1) mutation, missense mutation of progesterone receptor membrane component 1 (PGRMC1) and luteinizing hormone (LH) receptor mutation (LHR) [29]. Mutations of oocyte genes essential for meiosis and DNA repair have been also found in patients with POI, including synaptonemal complex protein 2 (SYCP2), mini-chromosome maintenance (MCM) 8 and 9, DNA primase subunit 1 (PRIM1) and tousled-like kinase (TLK1) [29,35]. Loss-of-function mutations of transcriptional factors in primordial germ cells and oocytes have been identified, including folliculogenesis-specific bHLH transcription factor (FIGLA); tumor protein 63 (TP63); spermatogenesis- and oogenesis-specific basic helix–loop–helix transcription factors (SOHLH) 1 and 2; forkhead box O (FOXO) 2, 3 and 4; nuclear protein transcriptional regulator 1 (NUPR1); and steroidogenic factor-1 (SF1) [29,36,37,38]. Enzymes of different groups, such as A disintegrin and metalloproteinase with thrombospondin motifs (ADAMTS) 16 and 19, DNA polymerase gamma catalytic subunit 1 (POLG1) and caseinolytic mitochondrial matrix peptidase proteolytic subunit (CLPP), were also found to have mutations in POI patients. One of the crucial mechanisms of ovarian senescence and follicular depletion involves impaired telomere length and telomerase activity [39].

Autoimmune diseases are considered to be responsible for 4–30% of POI cases [40]. The most common of these are Hashimoto thyroiditis, Graves’ disease, Addison’s disease, diabetes type 1, celiac disease, myasthenia gravis, systemic lupus erythematosus and autoimmune polyglandular syndromes (APS) [41]. The autoimmune mechanisms involved in developing POI include lymphocytic oophoritis and the presence of anti-oocyte antibodies (AOAs). Lymphocytic oophoritis occurs in cases of mononuclear infiltration of ovarian theca cells, involving the development of follicles and the corpus luteum, with a predominance of T-lymphocytes. The inflammatory response affects ovarian steroid production and ovarian function [42,43]. AOAs can be detected in 24–73% of patients with POI and may appear years before POI diagnosis [41,44]. Moreover, there are some reports of POI developing after viral infections, including one case report of COVID-19-related POI and AOA formation after anti-HPV vaccination [45,46].

Iatrogenic POI is a consequence of gonadotoxic treatments, including chemotherapy and radiotherapy. Chemotherapy affects ovarian function by increasing follicular apoptosis, causing ovarian cortical fibrosis; damage to the ovarian vasculature; and premature activation, recruitment and destruction of follicles. Nevertheless, the final effect of chemotherapy depends on several factors, such as a patient’s age, chemotherapy regimen and doses of drugs administered in the treatment [47]. Radiotherapy also has detrimental effects on the ovaries, as well as on the uterus and the whole hypothalamic–pituitary–gonadal axis [48,49]. Women with a history of cancer treatment in childhood and adolescence have an increased risk of developing POI later in life [50].

Finally, there is some evidence of the influence of several environmental factors on the prevalence of POI, with cigarette smoking and recurrent viral infections being the factors most commonly associated with POI. It is postulated that environmental agents may impact the ovarian function via direct action on estrogen receptors, induction of oxidative stress and epigenetic modifications [51]. 

Etiology of POI is summarized in Table 1.

## 4. The Role of Noncoding RNAs in the Pathophysiology of POI

The number of studies concerning the role of ncRNA, especially miRNA, in a wide range of human diseases, including cancer, cardiovascular and neurological disorders, has increased rapidly in recent years [52,53,54,55,56]. Among the studies concerning POI are those performed on animal models, as well as those performed in vivo, evaluating ncRNA expression in biological material samples collected from patients [57,58].

In the study performed by Dang et al. in 140 women with POI and 140 age-matched controls of Han Chinese ancestry, 51 differentially expressed miRNAs were identified—22 upregulated and 29 downregulated. This study revealed that miR-22-3p was significantly downregulated in the plasma of POI patients compared to healthy controls [59]. It was also revealed in other studies that MiR-22-3p suppresses FSH secretion, promotes granulosa cells apoptosis and suppresses estrogen receptor 1 (ESR1) and phosphatases and tensin homolog (PTEN), which are potential POI candidate genes [14,33]. Chen et al. revealed that significantly upregulated expression of miR-146a in the plasma and ovarian granulosa cells in patients with POI, with the targets in interleukin-1 receptor-associated kinase (IRAK1) and tumor necrosis factor receptor-associated factor 6 (TRAF6). These findings suggest a promoting effect on granulosa cell apoptosis via the caspase cascade pathway [60]. MiR-146aC > G has been implicated in differential expression of FOXO3 and cyclin D2 (CCND2)—genes related with POI [61]. In another study, three miRNA polymorphisms (miR-145aC > G, miR-196a2T > C and miR-499A > G) were investigated in the Korean population. Although none of the three polymorphisms alone were associated with POI, gene–gene interactions between miR-146 and miR-196a may be involved in the disease development [62]. Additionally, the miR-449b rs10061133 AA polymorphism was revealed as a risk factor for POI [63]. Target genes of miR-449b include estrogen-receptor-associated transcription factor (E2F1), which is involved in regulation of ovarian steroidogenesis, as well as miR-320 [14,64]. Other miRNAs involved in the regulation of ovarian steroidogenesis are miR-133b, which downregulates forkhead box L2 (FOXl2) expression in human and mouse granulosa cells; and miR-132, which inhibits progesterone production by decreasing steroidogenic acute regulatory protein (StAR) and promoting expression of steroidogenesis enzymes 3β-hydroxysteroid dehydrogenase (HSD) and 20α-hydroxysteroid dehydrogenase (20α-HSD). StAR is a key regulator of the conversion of cholesterol to pregnenolone, whereas 3β-HSD and 20α-HSD are responsible for the production of biologically inactive 20α-hydroxyprogesterone [65]. Yang et al. revealed that miR-23a is significantly upregulated in the plasma of POI patients and may be crucial for apoptosis induction in granulosa cells by targeting X-linked inhibitor of apoptosis protein (XIAP) and caspase signaling [66]. Moreover, both miR-23a and miR-27a promote granulosa cell apoptosis by targeting others against decapentaplegic homolog 5, known as SMAD5, a member of the TGFβ family that is involved in cell signaling [67]. Dang et al. showed that miR-379-5p was overexpressed in granulosa cells of patients with POI. This miRNA inhibited granulosa cell proliferation and attenuated DNA repair efficiency via poly (ADP-ribose) polymerase 1 (PARP1) and X-ray repair cross-complementing 6 (XRCC6) pathways [68]. Another study revealed the upregulation of miR-127-5p in granulosa cells from patients with POI and simultaneous downregulation of high-mobility group box 2 (HMGB2) in the same cohort of cases. MiR-127-5p was also confirmed to attenuate DNA repair capability via HMGB2 in mouse ovary samples. Moreover, miR-127-5p was overexpressed in the plasma of POI patients, suggesting its promising value as a biomarker of the disease [69]. Li et al. suggested that miR-21 and its target Pellino-1 (Peli1) might be involved in the pathogenesis of autoimmune POI. In this study, serum miR-21 levels in POI patients were lower than in the control group, which was positively related with Peli1, anti-Müllerian hormone (AMH), estradiol (E_2_), and the size of the uterus and ovarian volume, and negatively related to FSH, LH and the number of immune parameters [70].

Animal models are also used to identify the molecular mechanisms involved in POI and to find targets for potential therapy. The most common are mouse and rat models, because these animals show a high degree of similarity with humans in relation to ovarian development and functions. Animal models of POI are generated using induction with different chemotherapeutic drugs [71]. Kuang et al. identified 63 miRNAs that were upregulated and 20 miRNAs that were downregulated in ovarian tissue samples from 4-vinylcyclohexene diepoxide (VCD)-induced rat POI in comparison to control ovarian tissues. Among the upregulated miRNAs were miR-151 and miR-672 targeting expression of tumor necrosis factor superfamily member 10 (TNFSF10) and fibronectin type III domain-containing 1 (FNDC1), which are known as regulators of cell apoptosis. Among the downregulated miRNAs were miR-29a and miR-144, which may trigger the expression of phospholipase A2 group IVA (PLA2G4A) involved in prostaglandin biosynthesis [72]. Ai et al. investigated a mouse model of POI induced by tripterygium glycosides (TGs) and found that TGs induces cytotoxicity in ovarian granulosa cells via the Hippo-yes-associated protein (YAP)/transcriptional coactivator with the PDZ-binding motif (TAZ) pathway. In this model, miR-181b, miR-15a and miR-30d were significantly upregulated in POI. Moreover, the overexpression of miR-15a inhibited the proliferation and growth of murine ovarian granulosa cells, as well as induced their senescence [73]. In another study of cyclophosphamide-induced POI in mice, it was found that overexpression of miR-15b induces POI by silencing the endogenous α-Klotho mRNA and stimulating the activity of the downstream transforming growth factor β1 (TGFβ1)/SMAD pathway [74]. It was also shown in cultured mouse granulosa cells that were incubated with increasing doses of cisplatin that miR-125a-5p induced apoptosis of granulosa cells by decreasing signal transducer and activator of transcription 3 (STAT3). Since STAT3 plays a role in many reproductive functions by transducing signals in response to growth factors and cytokines, this finding provides new insights into the understanding of POI [75]. In a recently published report, Zhao et al. investigated the role of FK506-binding protein 4 (FKBP4), which is involved in the immunoregulation of lymphocytes B and T and in the regulation of steroid hormone receptor signaling, in a cisplatin-induced rat POI model. They found that FKBP4 ovarian expression was significantly decreased in POI and confirmed that this protein is a target of miR-483-5p. Upregulation of miR-483-5p increases ovarian sensitivity to cisplatin and causes severe ovarian dysfunction [76]. Additionally, high-fat and high-sugar diet supplementation was revealed to activate the Dab2ip/Ask1/p38-Mapk signaling pathway and to promote gamma H2A histone family member X (γH2AX) phosphorylation by inhibiting the expression of endogenous miR-146b-5p, which results in granulosa cell ageing and POI development [77]. The most common miRNAs involved in the pathophysiology of POI are summarized in Table 2.

In addition to miRNAs, there are also studies available regarding other ncRNA classes in POI [78]. Zhou et al. revealed different expression profiles of circRNAs in patients with biochemical POI, identifying a total of 133 upregulated and 424 downregulated circRNAs in POI patients in comparison to healthy controls. Among them, has_circ_003785 and has_circ_103903 were positively correlated with the basal FSH level, whereas has_circ_008389 was positively correlated with the AMH and antral follicle count (AFC). Additionally, the authors constructed circRNA–miRNA networks and found that the FoxO signaling pathway and cellular senescence were the most predominantly enriched signaling pathways [22]. Elizur et al. measured the transcripts levels of long noncoding RNA’s FMR4 and FMR6 in granulosa cells of FMR1 premutation carriers undergoing IVF treatment and pre-implantation genetic diagnosis (IVF-PGD) in comparison to women undergoing IVF treatment for male factors. There was a significant nonlinear association between the number of CGG repeats and the levels of FMR6 transcripts, and additionally, a significant negative linear correlation between the number of oocytes retrieved and the lncRNA levels in granulosa cells of FMR6. These findings suggest RNA toxic gain-of-function as one of the possible pathophysiologic mechanisms underlying FMR1-associated POI [34,79]. Wang et al. revealed downregulation of lncRNA HLA complex P5 (HCP5) in granulosa cells from women with POI. Functional experiments revealed a regulatory role of HCP5 in MutS homolog 5 (MSH5) expression via Y-box binding protein 1 (YB1) to affect the DNA damage repair progress of granulosa cells. MSH5 is a member of the mutS family of proteins that are involved in DNA mismatch repair or meiotic recombination processes, while causative mutations of MSH5 gene have been reported in women with POI. HCP5 silencing affected the localization of YB1 into the nucleus and reduced the binding of YB1 to the promoter of the MSH5 gene, diminishing MSH5 expression [80]. A recently published study also identified that hypermethylation-induced downregulation of lncRNA plasmacytoma variant translocation 1 (PVT1) promotes granulosa cell apoptosis in POI by inhibiting foxO3a phosphorylation and increases the FoxO3a transcription activity [81]. It was also very recently shown that lncRNA LINC02690, also called granulosa-cell-associated transcript 1 (GCAT1), is downregulated in granulosa cells from patients with biochemical POI. In their study, the authors also presented a significant correlation between downregulated GCAT1 and serum levels of follicle-stimulating hormone and anti-Müllerian hormone. Subsequent functional experiments suggested that downregulation of GCAT1 under conditions of POI inhibits the proliferation of granulosa cells through polypyrimidine tract-binding protein 1 (PTBP1)-dependent p27 regulation, which points to a novel form of lncRNA-mediated epigenetic regulation of granulosa cell function that contributes to the pathogenesis of POI [82]. The same group of researchers also found that another lncRNA, namely, ZNF674-AS1, is downregulated in granulosa cells from patients with POI, and its expression correlates with serum levels of clinical ovarian reserve indicators. As ZNF674-AS1 is induced by energy stress and regulates the proliferation and glycolysis of granulosa cells, it may possibly lead to follicular dysfunction through reduced enzymatic activity of aldolase A (ALDOA). This very recent study identified a new lncRNA–ALDOA complex through which ZNF674-AS1 exerts its functions, in line with a previous study contributing to our understanding of epigenetic regulation of granulosa cell function [83].

## 5. The Role of Noncoding RNAs in Potential Treatment of POI

Currently, there is no effective therapy for POI that is widely available in clinical practice, meaning once the ovarian function is lost, it cannot be returned. As such, the treatment of patients with POI is based on hormone replacement therapy, which aims to avoid severe health complications related to the lack of estrogens [3]; however, intensive research on human folliculogenesis and reproductive biology has led to the development of novel promising strategies, such as in vitro activation (IVA).

IVA is a method established by Kawamura and his team, which is based on the finding that even in patients with POI in ovarian tissue, some primordial, primary and secondary follicles are present that can be activated via two important signaling pathways, namely, the phosphoinositide 3-kinase(PI3K)/protein kinase B (Akt)/FOXO3 signaling pathway and the Hippo signaling pathway [84,85]. During the IVA procedure, ovarian cortex samples obtained from patients through laparoscopic surgery are fragmented into small cubes (approximately 1–2 mm^3^) to disrupt Hippo signaling. Then, the cubes are incubated with a PTEN inhibitor or a PI3K stimulator for 2 days followed by autotransplantation beneath the serosa of the Fallopian tubes. The follicle growth is then stimulated by exogenous gonadotropin under suppression of luteinizing hormone to generate competent mature oocytes for subsequent IVF procedure [86]. So far, at least two live births in 20 POI patients have been reported after IVA [86,87]. Recently, the IVA procedure was modified into so-called “drug-free IVA” to avoid the need for two surgical procedures. This approach is based only on tissue fragmentation and mechanical manipulation to disrupt the ovarian Hippo signaling pathway and promote follicle growth, and seems to be effective in patients with diminished ovarian reserves [88].

Other promising therapeutic strategies for POI patients are related to the use of exosomal miRNA derived from mesenchymal stem cells (MSC). MSC are a type of adult stem cells, which can be harvested from different tissues and fluids, such as bone marrow, umbilical cord, endometrial tissue, adipose tissue and amniotic fluid. With the capacity for self-renewal and differentiation potential, MSC are candidates for cell therapy in regenerative medicine, with many trials confirming their efficacy in a wide range of diseases, including cardiovascular disorders, diabetes, neurological diseases, renal fibrosis and female reproductive disorders [89,90,91]. Exosomes are a type of extracellular vesicles (EVs) secreted by cells in the extracellular space in response to different stimuli, both in physiological and pathological circumstances. They contain important materials, including DNAs, RNAs proteins and lipids, and have the ability for exchange between cells. Mesenchymal stem cell-derived exosomes (MSC-EVs) appear to have therapeutic effects on the granulosa cells in ovaries by promoting angiogenesis, regulating immunity and reducing oxidative stress [91,92]. Xiao et al. demonstrated that amniotic fluid stem cell (AMSC)-derived exosomes prevent ovarian follicular atresia in chemotherapy-treated mice via the delivery of miR-146a and miR-10a, with potential target genes being critical to apoptosis [93]. Zhang et al. revealed that human amniotic epithelial cell (hAEC) exosomes inhibited chemotherapy-induced granulosa cell apoptosis in mice by transferring functional miRNAs, such as miR-1246 [94]. Another study indicated that miR-644-5p carried by bone marrow MSC (BMSC)-derived exosomes inhibited the apoptosis of ovarian granulosa cell by targeting p53, suggesting that miR-644-5p has the potential to treat POI and restore ovarian function [95]. Additionally, miR-144-5p from BMSC-derived exosomes inhibited apoptosis in granulosa cells via targeting PTEN, thereby improving ovarian function in a cyclophosphamide-induced rat POI model [96]. MSC-EVs therapy influences not only granulosa cell apoptosis, but also mitigates oxidative stress—one of the crucial processes in ovarian depletion. Ding et al. demonstrated that exosomal miR-17-5p derived from human umbilical cord MSC (hUMSCs) restores ovarian function in chemotherapy-induced POI, alleviating oxidative stress via the inhibition of the SIRT7 and expression of its downstream target genes (PARP1, γH2AX and XRCC6) [97]. Sirtuins (SIRT1 to SIRT7) are major regulators of cellular responses to metabolic and oxidative stress, with SIRT7 deficiency contributing to accelerated cellular aging and defective embryogenesis [98]. As mentioned before, PARP1 and XRCC6 are critical mediators of DNA repair, whereas γH2AX is involved not only in DNA repair, but also in stem cell self-renewal and ageing [97,99]. In another study, the same authors illustrated the therapeutic effect of exosomal miR-320a released from human AMSCs in an animal model of POI. This effect was based on the prevention of reactive oxygen species generation by regulating SIRT4 [100]. The most common exosomal miRNA candidates for POI treatment are summarized in Table 3.

## 6. Summary

This review summarizes the recent advances in the understanding of the roles of noncoding RNAs, especially miRNAs, in the pathophysiology and potential treatment of POI. A full explanation of the molecular mechanism involved in the development of the disease is required in order to find proper, specific targets for effective treatment. In particular, the use of MSC-derived exosomes seems to be very promising; however, the available studies in this rapidly expanding field have been performed in animal models and still raise concerns regarding the acquisition, transportation and storage of MSC-derived exosomes, as well as difficulties regarding the commercialization and safety of this method.

## Figures and Tables

**Table 1:** Etiology of POI.

**Genetic**	Turner syndrome Fragile X syndrome (FMR1 premutation) Mutations in reproductive ligands and their receptors: FSHR BMP15 GDF9 GREM1 PGRMC1 LHR Mutations of oocyte genes essential for meiosis and DNA repair: SYCP2 MCM8 and 9 PRIM1 TLK1 Loss-of-function mutations of transcriptional factors in primordial germ cells, oocytes and granulosa cells: FIGLA TP63 SOHLH1 and 2 FOXO2, 3 and 4 NUPR1 SF1 Mutations of enzymes: ADAMTS16 and 19 POLG1 CLPP
**Autoimmune**	Hashimoto thyroiditis Graves’ disease Addison’s disease diabetes type 1 celiac disease myasthenia gravis systemic lupus erythematosus autoimmune polyglandular syndromes viral infections: HIV, COVID-19
**Iatrogenic**	Oncological treatment: chemotherapy and radiotherapy Surgical trauma (e.g., fulguration or cautery injury during excision of endometrioma)
**Environmental**	Cigarette smoking

FSHR = FSH receptor; BMP15 = bone morphogenic protein 15; GDF9 = growth differentiation factor 9; GREM1 = gremlin 1; PGRMC1 = progesterone receptor membrane component 1; LHR = LH receptor; SYCP2 = synaptonemal complex protein 2; MCM8 and 9 = mini-chromosome maintenance 8 and 9; PRIM1 = DNA primase subunit 1; TLK1 = tousled-like kinase; FIGLA = folliculogenesis-specific bHLH transcription factor; TP63 = tumor protein 63; SOHLH = spermatogenesis- and oogenesis-specific basic helix–loop–helix transcription factors 1 and 2; FOXO = forkhead box O; NUPR1 = nuclear protein transcriptional regulator 1; SF1 = steroidogenic factor-1; ADAMTS = A disintegrin and metalloproteinase with thrombospondin motifs; POLG1 = DNA polymerase gamma catalytic subunit 1; CLPP = caseinolytic mitochondrial matrix peptidase proteolytic subunit.

**Table 2:** The most common miRNAs involved in the pathophysiology of POI.

miRNA	Target Genes	Study
miR-151 miR-672	TNFSF10 FNDC1	[72]
miR-22-3p	ESR1 PTEN	[59]
miR-146a	IRAK1 TRAF6 FOXO3 CCND2	[60] [61]
miR-449b miR-320	E2F1	[63,64]
miR-133b	FOXl2	[65]
miR-132	StAR, 3βHSD, 20αHSD	[65]
miR-23a	XIAP	[66]
miR-23a miR-27a	SMAD5	[67]
miR-379-5p	PARP1 XRCC6	[68]
miR-127-5p	HMGB2	[69]
miR-21	Peli1	[70]
miR-29a miR-144	PLA2G4A	[72]
miR-15b	αKlotho mRNA TGFβ/SMAD pathway	[74]
miR-483-5p	FKBP4	[76]
miR-146b-5p	Dab2ip/Ask1/p38-MAPK γH2AX	[77]
miR-125a-5p	STAT3	[75]

TNFSF10 = tumor necrosis factor superfamily member 10; FNDC1 = fibronectin type III domain containing 1; ESR1 = estrogen receptor 1; PTEN = phosphatases and tensin homolog; IRAK1 = interleukin-1 receptor-associated kinase; TRAF6 = tumor necrosis factor receptor-associated factor 6; FOXO3 = forkhead box O; CCND2 = cyclin D2; E2F1 = estrogen receptor-associated transcription factor; FOXl2 = forkhead box L2; StAR = steroidogenic acute regulatory protein; 3βHSD = 3β-hydroxysteroid dehydrogenase; 20αHSD = 20α-hydroxysteroid dehydrogenase; XIAP = X-linked inhibitor of apoptosis protein; SMAD5 = others against decapentaplegic homolog 5; PARP1 = poly (ADP-ribose) polymerase 1; XRCC6 = X-ray repair cross-complementing 6; HMGB2 = high-mobility group box 2; Peli1 = Pellino-1; PLAG24A = phospholipase A2 group IVA; TGFβ = transforming growth factor β; FKBP4 = FK506-binding protein 4; γH2AX = gamma H2A histone family member X; Dab2ip = DAB2 interacting protein; Ask1 = apoptosis signal-regulating kinase 1; p38-MAPK = p38 mitogen-activated protein kinase; STAT3 = signal transducer and activator of transcription 3.

**Table 3:** The most common exosomal miRNA candidates for POI treatment.

miRNA	Source	Study
miR-146a miR-10a	AMSC	[93]
miR-1246	AEC	[94]
miR-644-5p	BMSC	[95]
miR-144-5p	BMSC	[96]
miR-17-5p	UMSC	[97]
miR-320a	AMSC	[100]

AMSC = amniotic fluid mesenchymal stem cells; AEC = amniotic epithelial mesenchymal stem cells; BMSC = bone marrow mesenchymal stem cells; UMSC = umbilical cord mesenchymal stem cells.

## Data Availability

No new data were created or analyzed in this study. Data sharing is not applicable to this article.
